# Emergence, Development, and Maturity of the Gonad of Two Species of Chitons “Sea Cockroach” (Mollusca: Polyplacophora) through the Early Life Stages

**DOI:** 10.1371/journal.pone.0069785

**Published:** 2013-08-02

**Authors:** Omar Hernando Avila-Poveda, Quetzalli Yasú Abadia-Chanona

**Affiliations:** 1 División de estudios de posgrado, Universidad del Mar, (UMAR), Puerto Ángel, Oaxaca, México; 2 Laboratorio de histología, Universidad del Mar (UMAR), Puerto Ángel, Oaxaca, México; Ecole Normale Supérieure de Lyon, France

## Abstract

This study describes and recognises, using histological and microscopical examinations on a morphometrical basis, several gonad traits through the early life stages of *Chiton articulatus* and *C. albolineatus*. Gonadal ontogenesis, gonad development stages, sexual differentiation, onset of the first sexual maturity, and growth sequences or “early life stages” were determined. In addition, allometry between lengths and body weight pooled for both sexes per each chiton were calculated using equation *Y = aX^b^*. A total of 125 chitons (4≤TL≤40 mm, in total length “TL”) were used. All allometric relations showed a strong positive correlation (*r*), close to 1, with b-values above three, indicating an isometric growth. Gonadal ontogenesis and gonad development stages were categorised into three periods (“Pw” without gonad, “Pe” gonad emergence, and “Pf” gonadal sac formed) and four stages (“S0” gametocytogenesis, “S1” gametogenesis, “S2” mature, and “S3” spawning), respectively. Compound digital images were attained for each process. Periods and stages are overlapped among them and between species, with the following overall confidence intervals in TL: Pw 6.13–14.32 mm, Pe 10.32–16.93 mm, Pf 12.99–25.01 mm, S0 16.08–24.34 mm (females) and 19.51–26.60 mm (males), S1 27.15–35.63 mm (females) and 23.45–32.27 mm (males), S2 24.48–40.24 mm (females) and 25.45–32.87 mm (males). Sexual differentiation (in S0) of both chitons occurs first as a female then as a male; although, males reach the onset of the first sexual maturity earlier than females, thus for *C. articulatus* males at 17 mm and females at 32 mm, and for *C. albolineatus* males at 23.5 mm and females at 28 mm, all in TL. Four early life stages (i.e., subjuvenile, juvenile, subadult, and adult) are described and proposed to distinguish growth sequences. Our results may be useful to diverse disciplines, from developmental biology to fisheries management.

## Introduction

In invertebrates, the origin and development of the gonad is complex and varied, and it is scarcely described in the literature even though it is a fundamental aspect of developmental biology. The reproductive anatomical features (gonad and gonoducts) of polyplacophorans have been studied in a wide spectrum of species around the world because of their relevance to phylogeny. In essence, these studies have targeted the morphography by light and/or electron microscopy of gonad structure and the shape of gametes but always of adult chitons [1]–[8], with special regard to: accumulation of substances such as nucleic acids, proteins, and mucopolysaccharides during oocyte differentiation [9]–[13]; follicle cells associated with the oocyte surface [14]–[18]; the chorion (also called hull) of the mature ovum surface [19]–[24]; spermiogenesis and acrosome formation [25]–[27]; and even the general aspect of both female and male gametes in the same gonad of a hermaphrodite chiton [28]–[29], among others.

In contrast, the origin and/or the development and/or maturation of the gonads of chitons through early life stages have been described to a much lesser extent, these studies being from the early 20th century (i.e., 1898–1912). Plate [30]–[32] published several treatises on the general anatomy of Chilean chitons, including microscopic traverse sections showing the origin and development of the gonad. Haller [33] described the coelom of chitons including an ovary and a testis, each beginning the emergence of gametogonia (i.e., spermatogonia and oogonia), that is, sexual differentiation. Heath [34] indicated that sexual differentiation occurs first as a female then as a male during the early stages of the chiton *Trachydermon raymondi*, when the ova appear in a typical fashion, but as the chiton grows, some of the primitive sex cells on the wall of the gonad commence to divide rapidly and ultimately form small clusters of spermatozoa among the neighbouring ova. Higley [35] described the gross anatomy of gonad development during the early life of chitons *Trachydermon raymondi* and *Nuttallina thomasi*.

Almost a century has elapsed (until 1974–1999) and similar studies were published, such as Richter [36], who described histologically the onset of the first sexual maturity in *Lepidochitona cinerea* (as *L. cinereus*). Ball [37] identified the smallest male and female chiton, as well as the onset of the first sexual maturity of *Stenoplax conspicua*. Otway [38] indicated that the chitons *Onithochiton quercinus* and *Plaxiphora albida* reached the onset of the first reproductive maturity at 2 years of age, i.e., when they still have a medium size as compared to adult size.

Even though there is an extensive knowledge of the gonad of chitons, in both adults and non-adults, this knowledge is not integrated making it difficult to determine the gonadal ontogeny (origin, development and maturation) of chitons through their early life stages, which is one of the fundamental aspects of developmental biology. However, some generalizations can be drawn. According to previous authors, the gonadal ontogenesis, the sexual differentiation and the onset of the first sexual maturity, apparently happens too early in the life cycle almost immediately after the metamorphic stage. However, information from other mollusks has indicated that they undergo many physiological, behavioural and digestive changes during this post-embryonic stage and/or post-hatching stage (post-metamorphic stage in chitons) before gonadal morphogenesis. So, the present study hypothesises that the origin, development and maturation of chitons gonads really happens just after the post-metamorphic stage (i.e., subjuvenile stage) perhaps during the juvenile stage; so it is also important to distinguish growth sequences (early life stages) based on a consistent terminology: subjuvenile “post-metamorphosed”, juvenile, subadult, and adult.

The aim of our investigation is to provide, on the basis of histological and microscopic examinations, descriptions on the emergence, development, and maturity of the gonad through the early life stages (i.e., from subjuvenile and along the juvenile and subadult stages) of two wild chitons of the Chitonidae family, *Chiton articulatus* and *Chiton albolineatus*. Likewise, we aimed to distinguish qualitatively and quantitatively these early life stages based on morphometry, as well as recognise the size range at which chitons display discernible gonads (gonadal differentiation), and the onset of the first sexual maturity (and not referring to the size at which 50% of a population is sexual mature given that we are not evaluating an adult population). In addition, allometric relationships between the lengths and body weight of chitons throughout early life stages are evaluated, because chitons subtly change their body shape as they develop into adulthood but particularly evolve in response to gonad maturation. This basic information may be useful to diverse disciplines, from developmental biology to fisheries management, since recently these two chitons [but particularly *Chiton articulatus* (locally known as “dog tongue” or “sea cockroach”)] have gained regional importance and economic interest in the southern Mexican Pacific, where some restaurants have begun to offer the “sea cockroach” to tourists as a gourmet and aphrodisiac food ([39]–[42], Avila-Poveda and Abadia-Chanona, pers. obs.).

## Materials and Methods

### Ethics statement

No specific legal permits were required for the collection of the specimens in the sampling location, because the location is not privately owned or a protected area in any way. Besides, the collections did not involve endangered or protected species. Both collected species have no current regulatory body concerned with the protection of wildlife or fishery management.

### Collections

**Figure 1:**
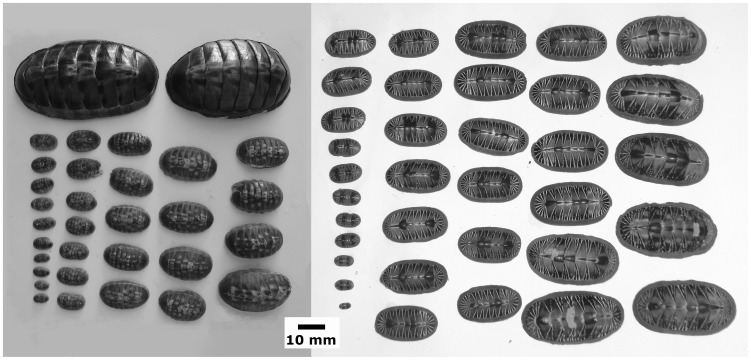
*Chiton articulatus* and *Chiton albolineatus* preserved. *Chiton articulatus* (left) and *Chiton albolineatus* (right) preserved, showing some of the several sizes here studied.

During the collection of *Chiton articulatus* adults in Oaxaca [43], *Chiton articulatus* and *Chiton albolineatus* of different sizes (i.e., subjuvenile “post-metamorphosed”, juvenile, subadult, and adult) were detected near high tide and on exposed rocks. Hence, along the rocky intertidal shores of the town of Puerto Angel (15°39′N–96°29′W), *C. articulatus* (*n* = 72 sampling size, with total length “TL” of 5≤TL≤40 mm) were collected during April and May of 2011, and *C. albolineatus* (*n* = 53, 4≤TL≤40 mm) was collected during March of 2012 (Fig. 1). The smallest adult size for both collected chitons was established at 40 mm TL, based on the modal size-class from other Mexican populations of *C. articulatus* [44]–[46] and *C. albolineatus* [47].

### Histological procedure and measurements

Chitons were relaxed and allowed to extend for 1 h, with gradual additions of tap water to the seawater, until the salinity reached half the salinity of the seawater of the sampling site (i.e., 50∶50 in volume); and this relaxation process was accelerated by adding a few milliliters of 10% ethanol [43], [48]. Later, chitons were fixed in 10% neutral formalin-saline solution in seawater for two weeks. Subsequently, they were preserved in 70% ethanol with 0.1% glycerin until histological processing [49]–[50]. After preservation, all chitons collected were weighed with an analytical balance (±0.001 g, body weight “BW”) and measured with a vernier caliper (±0.1 mm, total length “TL” including the mantle girdle, and total width “TW” including the mantle girdle). Weight lost at the end of preservation was estimated at a 2% loss of live weight [43]. Smaller chitons (TL≤15 mm) were decalcified for one week, immersed in 5% commercial vinegar (glacial acetic acid in water), and the others were disarticulated, valve by valve, keeping the gross soft body intact and complete.

**Figure 2:**
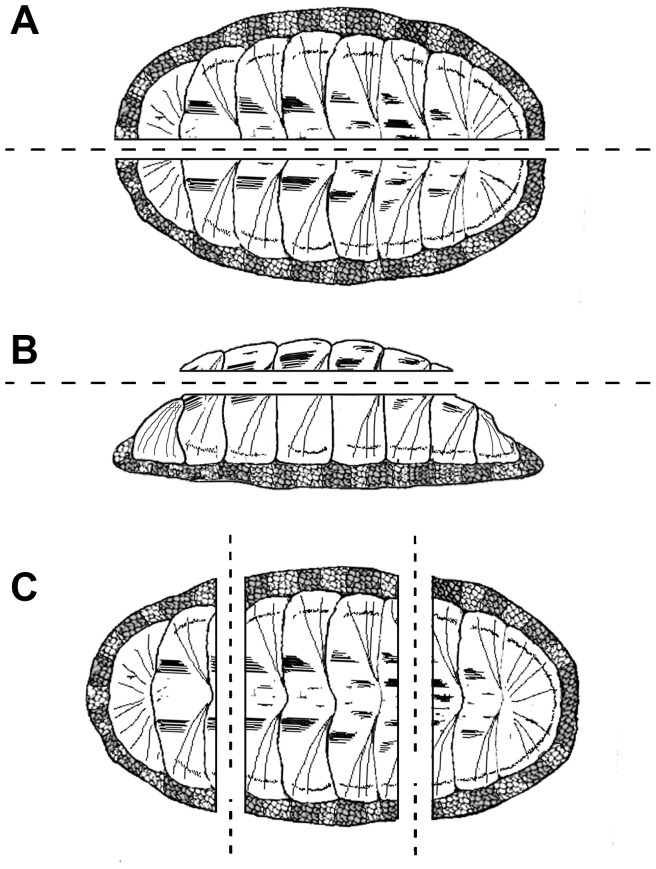
Diagram of *Chiton articulatus* illustrating the location and orientation of the cutting planes. (**A**) Dorsoventral orientation to permit the cutting of sagittal sections. (**B**) Lateral orientation to permit the cutting of frontal sections. (**C**) Dorsoventral orientation to permit the cutting of several transverse sections.

Chitons were haphazardly cut along the total length (TL) in sagittal (dorsoventral orientation) and frontal (lateral orientation) planes, as well as along the total width (TW) in several transverse planes (Fig. 2). Each portion was dehydrated in an ethanol series, cleared in Citrisolv®, and infiltrated and embedded in paraffin [50]–[51]. Serial sections were cut at 5-µm thickness using a manual rotary microtome (LEICA® RM2145) and mounted on glass slides. Groat's hematoxylin and erythrosine was used as basic stain since it gives better contrast [52]–[53]. The modified Crossmon's trichrome method (i.e., Groat's haematoxylin, erythrosine B-Orange G, and trypan blue and/or light green [54]–[56]) was used to contrast connective tissue, as well as to reveal vitelline reserves (i.e., yolk granules) and vacuolar processes.

### Microscopic examination

Here, two levels of formation and development of gonads were followed: 1) emergence and formation of the gonadal sac (i.e., gonadal ontogenesis) and, later, 2) the gonad development stages (i.e., changes in the ovary and/or testis during growth and maturation), respectively: 1) periods and 2) stages. Periods (P) of gonadal sac formation were assigned based on descriptions given for other species of chitons [6], [32]–[35], using the letter in lowercase of the representative process to give the period's name: Pw: without gonad, Pe: gonad emergence, and Pf: gonadal sac formed. Stages (S) of gonad development were classified based on the sequence of the development process of sexual cells (i.e., gametes) of female chitons [1], [2], [4], [14]–[17], [36] and male chitons [25]–[27]; thereby, for both sexes: S0: gametocytogenesis (i.e., immature gametes), S1: gametogenesis, S2: mature, and S3 spawning. Nonetheless, the S3 spawning (an adult stage) was not observed and is only marked in some graphs to understand better the developmental process of sexual cells (i.e., gametes) in the context of early life stages.

The photomicrographs were obtained with a digital camera (Sony Cybershot DSC-W520, 14.1 Mpixels) mounted on a microscope (Olympus CX21-FS1), and were stored in a computer in graphic format. Adobe Photoshop® CS2, version 9.0, was used to adjust the contrast and level of the images, for the photographic assembly process, and to produce composite images with numerous microscopic fields of 100× and 400× visual magnifications that show the gross microscopic features of chitons. General morphological terminology follows Richter [4], Plate [30]–[32], Haller [33], Higley and Heath [35], and Richter [36], among others. Each oocyte along its development was measured through sections containing the nucleus using Carl Zeiss Microimaging ® AxioVision Release, Version 4.8.2.0.

### Allometry and confidence intervals

In addition, the allometric relationships between the lengths (total length “TL”, total width “TW”) and body weight (BW), pooled for both sexes per chiton species were determined with reference to the equation *Y = aX^b^* [43], [57]–[58], where *b* (exponent) is of interest as it represents the growth type [59]. The levels (periods and stages) here described were also analysed using 95% confidence limits for the mean of the total length “TL” in order to estimate the intervals among these levels [60]. Each confidence interval was expressed as *P* (value mm≤*μ*≤value mm) [60]. Statistical analyses were carried out using the computer program STATISTICA® 6 and SigmaStat® 3.5.

## Results

### Allometry through the early life stages

**Figure 3:**
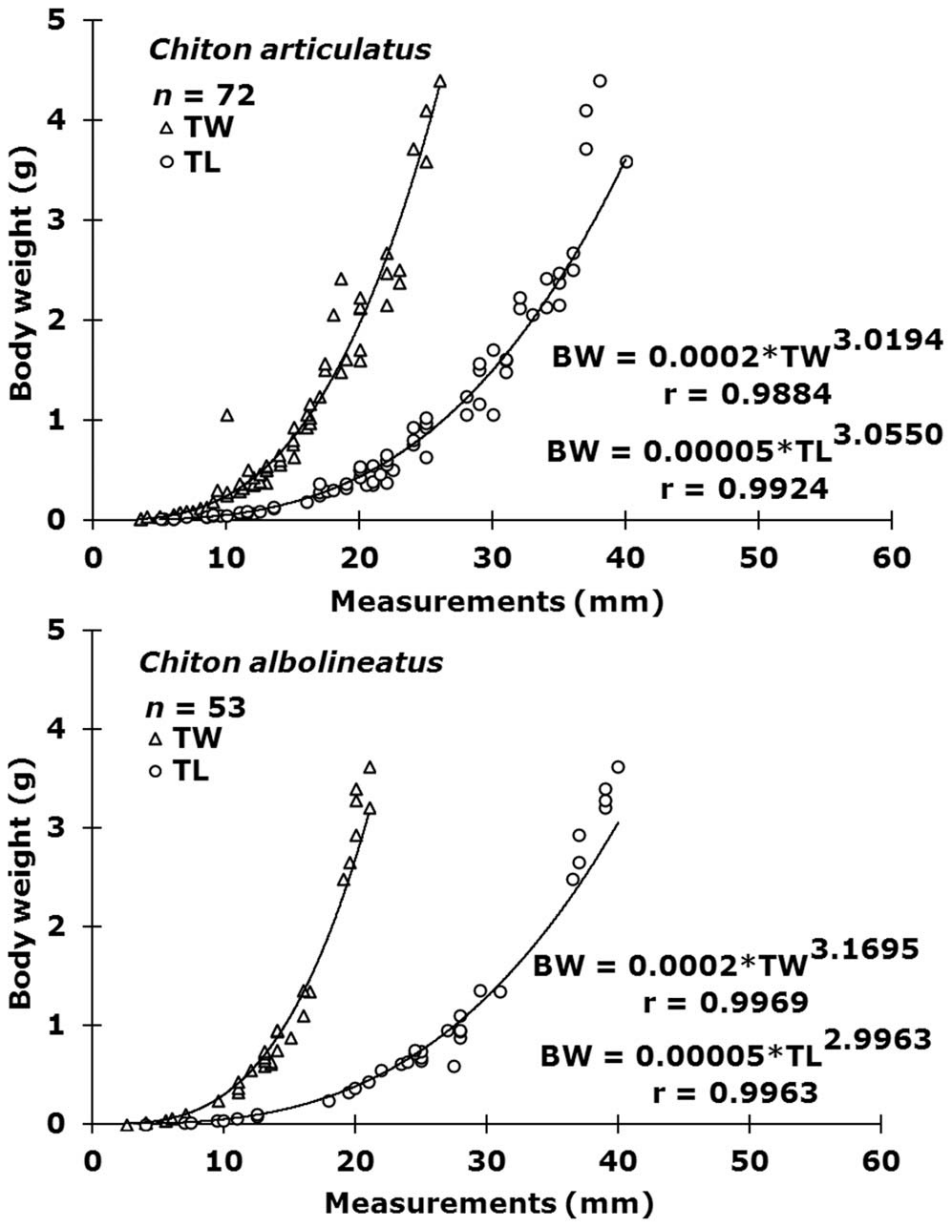
Allometric relationships during the early life of *Chiton articulatus* and *Chiton albolineatus*. Relationships between measurements (total weight “TW”, and total length “TL”) and body weight (BW) pooled for both sexes of *Chiton articulatus* (0.007 to 4.400 g BW) and *Chiton albolineatus* (0.004 to 3.632 g BW) during the range of early life here studied. Continuous line indicates equation tendency.

The relation between the lengths (total length “TL”, total width “TW”) and body weight (BW), pooled for both sexes per each chiton species during the early life stages (0.007 to 4.400 g BW in *Chiton articulatus*, and 0.004 to 3.632 g BW in *Chiton albolineatus*), showed an equation (*Y* = a*X* ^b^) of strong positive correlation (r) close to 0.99. The b-value ranged from 3.02 to 3.06 in *Chiton articulatus* and from 3.00 to 3.16 in *C. albolineatus* (Fig. 3).

### Gonadal ontogenesis

**Figure 4:**
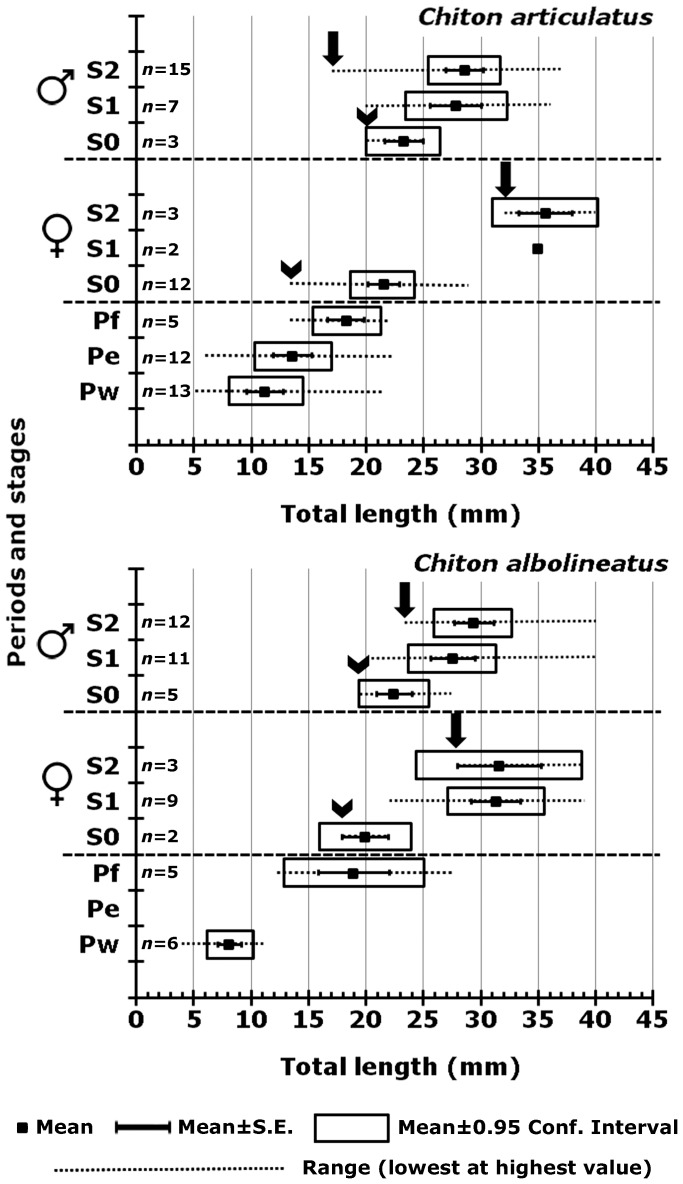
Ontogenesis and maturity of gonads through the early life of *Chiton articulatus* and *Chiton albolineatus*. Relation among total length (mm) and the periods of the gonadal sac formation (Pw: without gonad, Pe: gonadal emergence, Pf: gonadal sac formed), and the stages of gonad development (S0: gametocytogenesis, S1: gametogenesis, and S2: mature) in males and females from *Chiton articulatus* and *Chiton albolineatus*. The arrowhead indicates the sexual differentiation, and the arrow indicates the onset of the first sexual maturity. Number of data (*n*).

**Figure 5:**
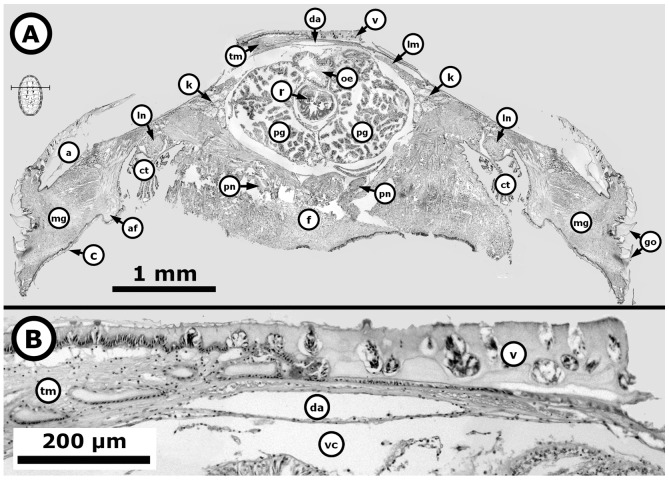
*Chiton* through the period without gonad. *Chiton articulatus* (0.074 g BW, 11 mm TL, and 7 mm TW) during the period without gonads. (**A**) Composite digital image in transverse section of the entire specimen showing the general histological anatomy. (**B**) Close-up of the dorsal part of a whole specimen. Abbreviations: a, articulamentum; af, accessory fold; c, cuticle; ct, ctenidium; da, dorsal aorta; f, foot; go, girdle ornamentations; k, kidney; lm, longitudinal muscle; ln, lateral “visceral” nerve cord; mg, mantle girdle; oe, oesophagus; pg, pharyngeal gland; pn, pedal nerve cord; r, radula; tm, transversal muscle; v, valve; vc, visceral cavity “hemocoel”.

The gonadal sac of chitons is a nearly round structure, but its shape and absolute size vary depending on the number and diameter of gametes enclosed. The formation of the gonadal sac takes place in three periods, as follows: without gonad (Pw), gonad emergence (Pe), and gonadal sac formed (Pf), and generally evolves with increasing total length (TL) of chitons, but each period is represented by a narrow range of total lengths with considerable overlap among periods. Thereby, the confidence interval in total length “TL” for the period without gonad (Pw) in *Chiton articulatus* was *P* (8.12 mm≤*μ*≤14.32 mm) and in *Chiton albolineatus* was *P* (6.13 mm≤*μ*≤10.20 mm) (Fig. 4, Pw), and qualitatively showed only the dorsal aorta covered by muscle tissue and connective fibres, without projections of blood sinus and blood vessels inward of the visceral cavity (Fig. 5).

**Figure 6:**
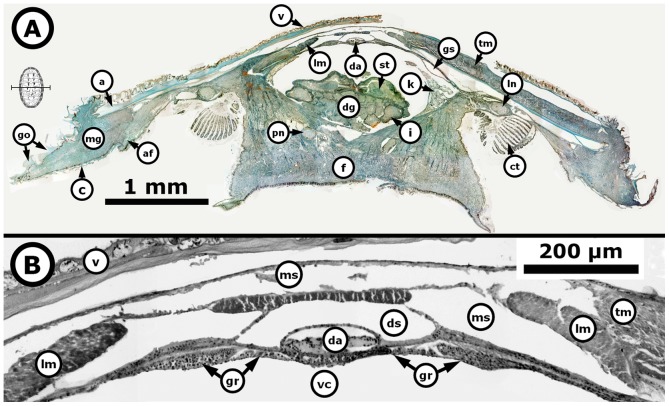
*Chiton* through the starting period of gonad emergence. *Chiton articulatus* (0.053 g BW, 9 mm TL, and 6 mm TW) through the starting period of gonad emergence, showing the projection of the squamous epithelium that extends along the wall of the dorsal aorta and is thrown toward the visceral cavity. (**A**) Composite digital image in transverse section of the whole specimen showing the general histological anatomy. (**B**) Close-up of the dorsal part from the whole specimen. Abbreviations: a, articulamentum; af, accessory fold; c, cuticle; ct, ctenidium; da, dorsal aorta; dg, digestive gland; ds, dorsal aorta sinus; f, foot; go, girdle ornamentations; gr, gonadal ridge (i.e., the precursor to the gonad); gs, gonadal sinus (i.e., cavity formed by a bending or curving from the gonadal ridge and containing chiefly blood); i, intestine; k, kidney; lm, longitudinal muscle; ln, lateral “visceral” nerve cord; mg, mantle girdle; ms, muscle sinus; pn, pedal nerve cord; st, stomach; tm, transversal muscle; v, valve; vc, visceral cavity “hemocoel”.

**Figure 7:**
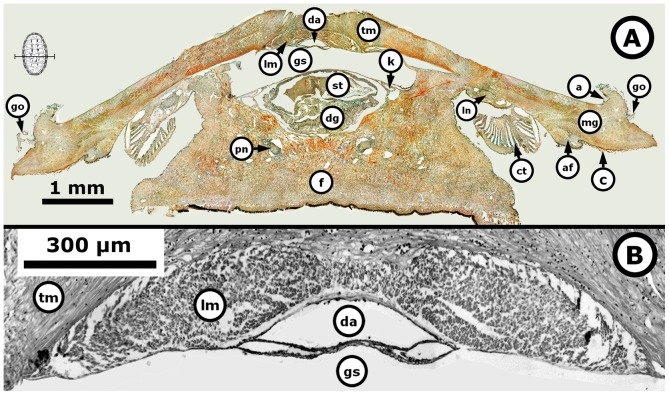
*Chiton* through the formation of the gonadal lobes. *Chiton articulatus* (0.382 g BW, 21 mm TL, and 12.5 mm TW) through the middle period of gonad emergence, showing the formation of two oval lobes. (**A**) Composite digital image in transverse section of the whole specimen showing the general histological anatomy. (**B**) Close-up of the dorsal part of the whole specimen. Abbreviations: a, articulamentum; af, accessory fold; c, cuticle; ct, ctenidium; da, dorsal aorta; dg, digestive gland; f, foot; go, girdle ornamentation; gs, gonadal sinus (i.e., cavity formed by a bending or curving from the gonadal ridge and containing chiefly blood); k, kidney; lm, longitudinal muscle; ln, lateral “visceral” nerve cord; mg, mantle girdle; pn, pedal nerve cord; st, stomach; tm, transversal muscle.

**Figure 8:**
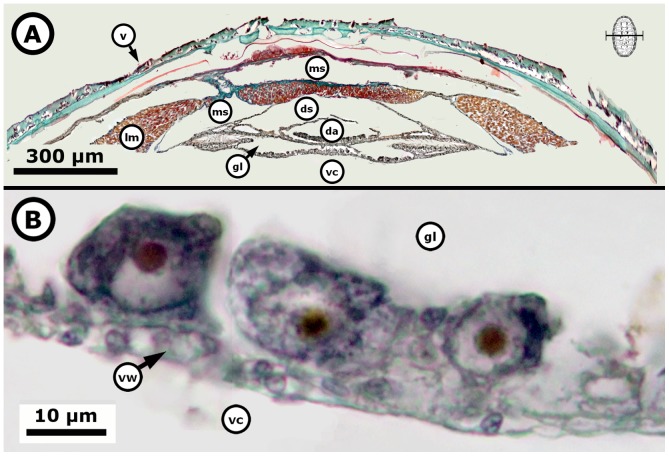
*Chiton* through the merging of the gonadal lobes. *Chiton articulatus* (0.185 g BW, 16 mm TL, and mm 9 TW) through the last period of gonad emergence, showing the ciliated cuboidal epithelial cells and the beginning of the merging of the lobes of the gonad sac. (**A**) Composite digital image in transverse section of the dorsal part of the whole specimen showing the general histological anatomy. (**B**) Close-up of the ventral right side of the gonad sac without tissue plates but showing the rise of the early oogonia with basophilic granular cytoplasm and one nucleolus, as well as the development of the early cells that constitute the tissue plates on the ventral wall of the gonad sac. Abbreviations: da, dorsal aorta; ds, dorsal aorta sinus; gl, gonadal lumen (i.e., an inner open space or cavity of the gonad), lm, longitudinal muscle; ms, muscle sinus; v, valve; vc, visceral cavity “hemocoel”; vw, ventral wall of gonad sac.

**Figure 9:**
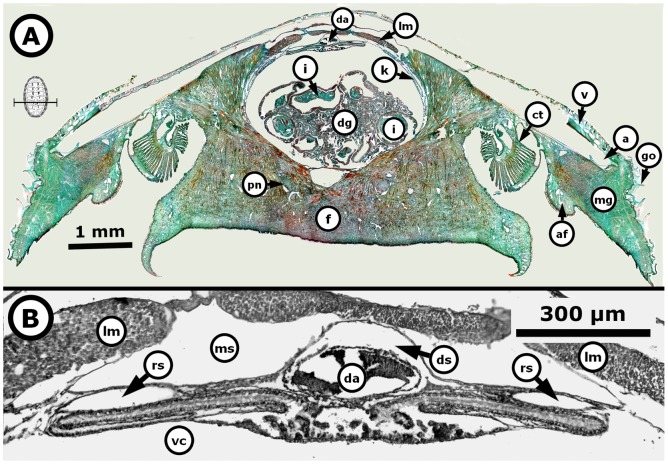
*Chiton* forming the gonad sac. *Chiton articulatus* (0.347 g BW, 21 mm TL, and 12 mm TW) through the last period of gonad emergence, showing the ciliated cuboidal epithelial cells, the merged lobes, the rise of the tissue plates and, in turn, development toward the gonad sac formed. (**A**) Composite digital image in transverse section of the whole specimen showing the general histological anatomy. (**B**) Close-up of the dorsal part of the whole specimen. Abbreviations: a, articulamentum; af, accessory fold; ct, ctenidium; da, dorsal aorta; dg, digestive gland; ds, dorsal aorta sinus; f, foot; go, girdle ornamentation; i, intestine; k, kidney; lm, longitudinal muscle; mg, mantle girdle; ms, muscle sinus; pn, pedal nerve cord; rs, residual gonadal sinus; v, valve; vc, visceral cavity “hemocoel”.

The period of gonad emergence (Pe) in *C. articulatus* started overlapping totally with Pw, i.e., with confidence interval in TL of *P* (10.32 mm≤*μ*≤16.93 mm); whilst in *C. albolineatus* this period of gonad emergence (Pe) was not observed (Fig. 4, Pe). The period of gonadal sac formed (Pf) in *C. articulatus* started very close to and overlapped with Pe, i.e., with confidence interval of *P* (15.21 mm≤*μ*≤21.39 mm), while in *C. albolineatus* it occurred without overlapping previous periods, i.e., with confidence interval of *P* (12.99 mm≤*μ*≤25.01 mm) (Fig. 4, Pf). Thus, the first appearance of the gonad evolves as a projection of squamous epithelium that extends from the wall of the dorsal aorta and is thrown toward the visceral cavity; this could be termed the “gonadal ridge” or the precursor to the gonad (Fig. 6). This squamous epithelium continues projecting until closing very narrowly against the dorsal aorta forming two oval lobes (Fig. 7). Later, the squamous epithelium from the wall of the dorsal aorta becomes ciliated cuboidal epithelial cells, and the two oval lobes, in turn, merge and develop into the gonad sac (Fig. 8). Finally, the built gonad sac starts to expand to form a hollow space (i.e., gonadal lumen), and in turn, the ventral wall of the gonad sac invaginates, forming folds inward of the lumen (i.e., tissue plates) (Fig. 9).

### Gonad development stages

#### Stage 0

**Figure 10:**
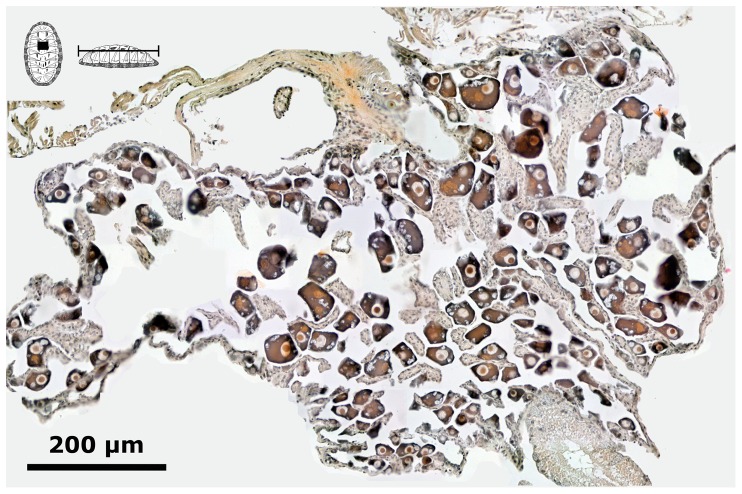
Gametocytogenesis in a *Chiton* female. Composite digital image in frontal section of the anterior dorsal part of a *Chiton articulatus* female (1.161 g BW, 29 mm TL, and 16.5 mm TW) with the ovary in the gametocytogenesis stage (S0) showing the formation of the primary oocytes.

**Figure 11:**
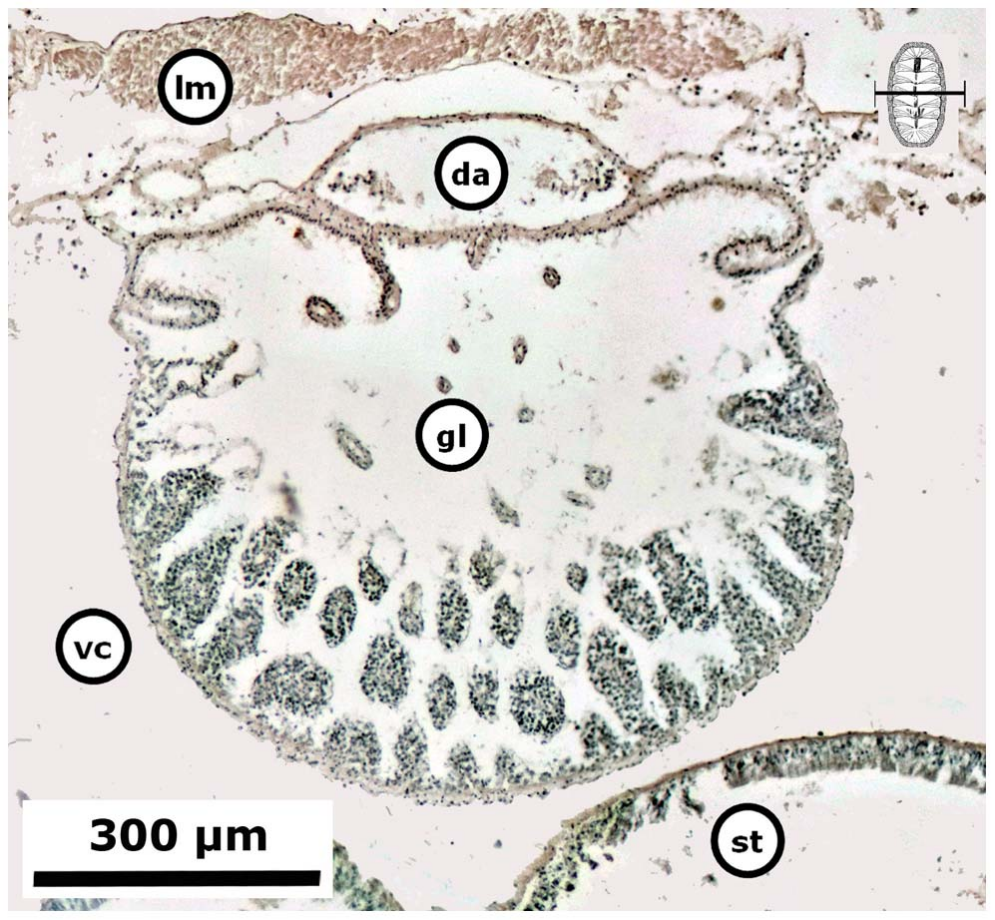
Gametocytogenesis in a *Chiton* male. Composite digital image in transverse section of a *Chiton albolineatus* male (0.596 g BW, 27.5 mm TL, and 13 mm TW) through the gametocytogenesis stage (S0), showing the general histological anatomy of the gonad sac wholly expanded forming a gonadal lumen and with several tissue plates occurring above the ventral wall. Early spermatocytes occur around the tissue plates. Abbreviations: da, dorsal aorta; gl, gonadal lumen, lm, longitudinal muscle; st, stomach; vc, visceral cavity “hemocoel”.

Gametocytogenesis (i.e., period of formation of early oocytes or early spermatocytes, i.e., immature gametes) takes place overlapping the periods of gonad emergence (Pe) and of gonadal sac formation (Pf) in both chiton species (Fig. 4). In females, the first sexual cells (i.e., oogonia and early oocytes) arise exceptionally early during the end of the gonad emergence period (Pe, Fig. 4), when the cuboidal epithelial cells of the gonadal sac are created and the two oval lobes have merged, but still without tissue plates (Fig. 8B); i.e., with a confidence interval of *P* (18.83 mm≤*μ*≤24.34 mm) for *C. articulatus* and of *P* (16.08 mm≤*μ*≤23.92 mm) for *C. albolineatus* (Fig. 4, S0-females). The primary oocytes measured from 15 to 62.5 µm in diameter and were characterised by a hyaline cytoplasm that was vacuolated and heavily basophilic, whereas the nucleus (germinal vesicle) was translucent with one basophilic spherical nucleolus (Fig. 10). In males, the first sexual cells (i.e., spermatogonia and early spermatocytes) arose later than in females during the end of the period of gonad sac formation (Pf, Fig. 4), when the gonad sac is wholly expanded forming a gonadal lumen, and several tissue plates occur above the ventral wall covered by early spermatocytes (Fig. 11); i.e., with confidence interval of *P* (20.07 mm≤*μ*≤26.60 mm) for *C. articulatus* and of *P* (19.51 mm≤*μ*≤25.49 mm) for *C. albolineatus* (Fig. 4, S0-males).

#### Stage 1

**Figure 12:**
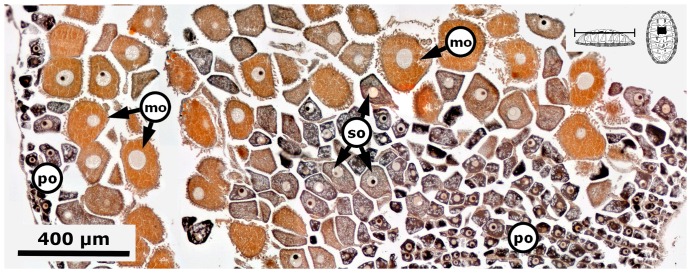
Gametogenesis in a *Chiton articulatus* female. Composite digital image in frontal section of the anterior dorsal part of a *Chiton articulatus* female (2.469 g BW, 35 mm TL, and 22 mm TW) with the ovary in the gametogenesis stage (S1) showing asynchronous groups of oocytes, i.e., primary oocytes, secondary oocytes, and mature ova without a dominant group. Abbreviations: po, primary oocytes; so, secondary oocytes; mo, mature ova.

**Figure 13:**
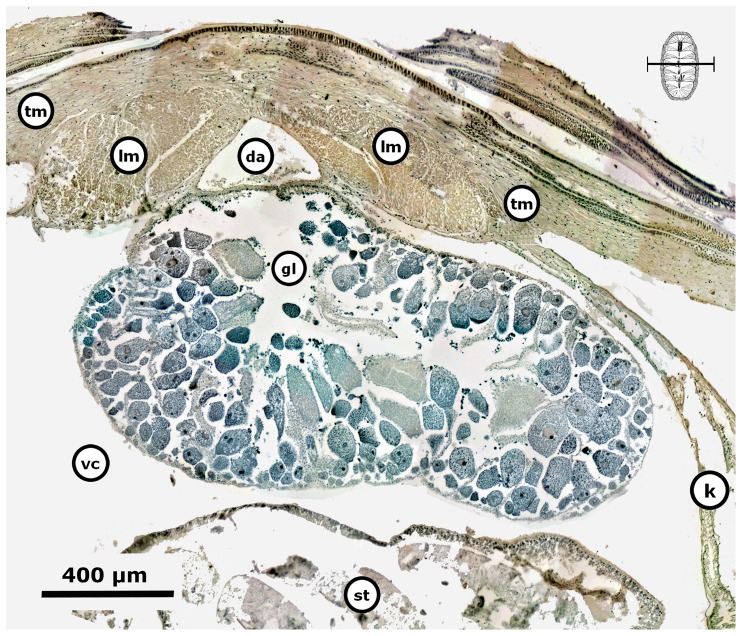
Gametogenesis in a *Chiton albolineatus* female. Composite digital image in transverse section of the dorsal part of a *Chiton albolineatus* female (0.953 g BW, 28 mm TL, and 14 mm TW) with the ovary in the gametogenesis stage (S1) showing only a synchronous group of secondary oocytes without replenishment by earlier oocytes. Abbreviations: da, dorsal aorta; k, kidney; gl, gonadal lumen; lm, longitudinal muscle; st, stomach; tm, transversal muscle; vc, visceral cavity “hemocoel”.

**Figure 14:**
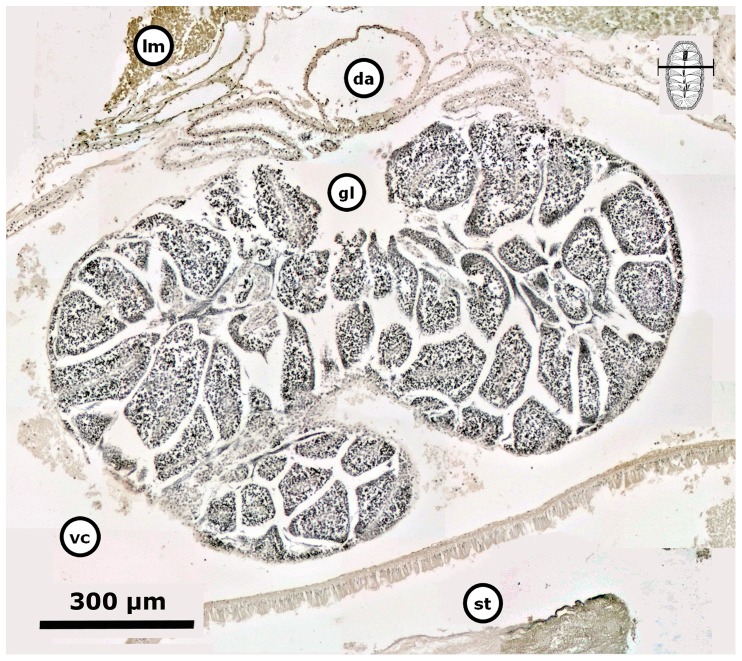
Gametogenesis in a *Chiton* male. Composite digital image in transverse section of a *Chiton albolineatus* male (0.434 g BW, 21 mm TL, and 11 mm TW) with testis in the gametogenesis stage (S1) showing spermatocytes and spermatids occurring around the tissue plates and forming groups, and some spermatozoa beginning to replenish the gonadal lumen. Abbreviations: da, dorsal aorta; gl, gonadal lumen, lm, longitudinal muscle; st, stomach; vc, visceral cavity “hemocoel”.

Gametogenesis, i.e., period of formation of secondary oocytes or secondary spermatocytes and spermatids. Females of *C. articulatus* showed asynchronous groups of oocytes, i.e., primary oocytes, secondary oocytes, and mature ova were present without a dominant group (Fig. 12), whereas *C. albolineatus* females showed only a synchronous group of secondary oocytes without replenishment by earlier oocytes (Fig. 13). This occurred around 35 mm in total length “TL” (*n* = 2) for *C. articulatus* and with a confidence interval of *P* (27.15 mm≤*μ*≤35.63 mm) for *C. albolineatus* (Fig. 4, S1-females). The secondary oocytes measured from 46 to 162 µm in diameter, and were characterised by a cytoplasm somewhat diminished of basophilia, nonvacuolated but wholly granular, with relatively smaller, spherical nucleus than early oocytes. Some secondary oocytes displayed or not follicle cells surrounding them (Fig. 12 and 13). Males in gametogenesis had a confidence interval in TL of *P* (23.45 mm≤*μ*≤32.27 mm) for *C. articulatus* and of *P* (23.89 mm≤*μ*≤31.38 mm) for *C. albolineatus* (Fig. 4, S1-males); showing a testis with spermatocytes and spermatids occurring around the tissue plates and forming groups, and some spermatozoa beginning to replenish the gonadal lumen (Fig. 14).

#### Stage 2

**Figure 15:**
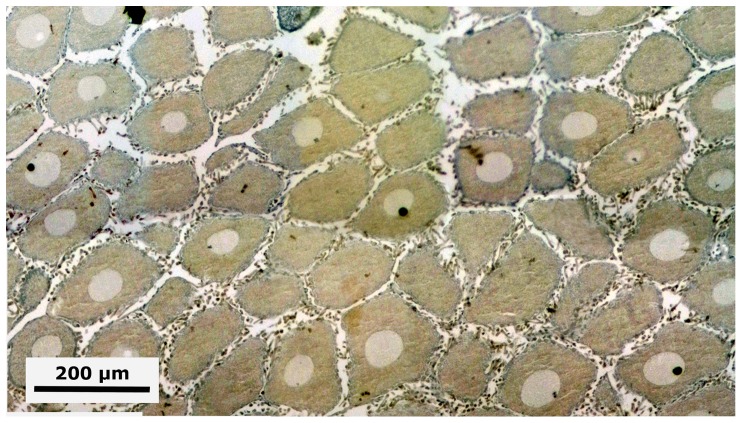
Mature *Chiton* female. Composite digital image in transverse section of an ovary of *Chiton articulatus* (2.228 g BW, 32 mm TL, and 22 mm TW) in mature stage (S2) showing only a synchronous group of mature ova without replenishment by earlier oocytes.

**Figure 16:**
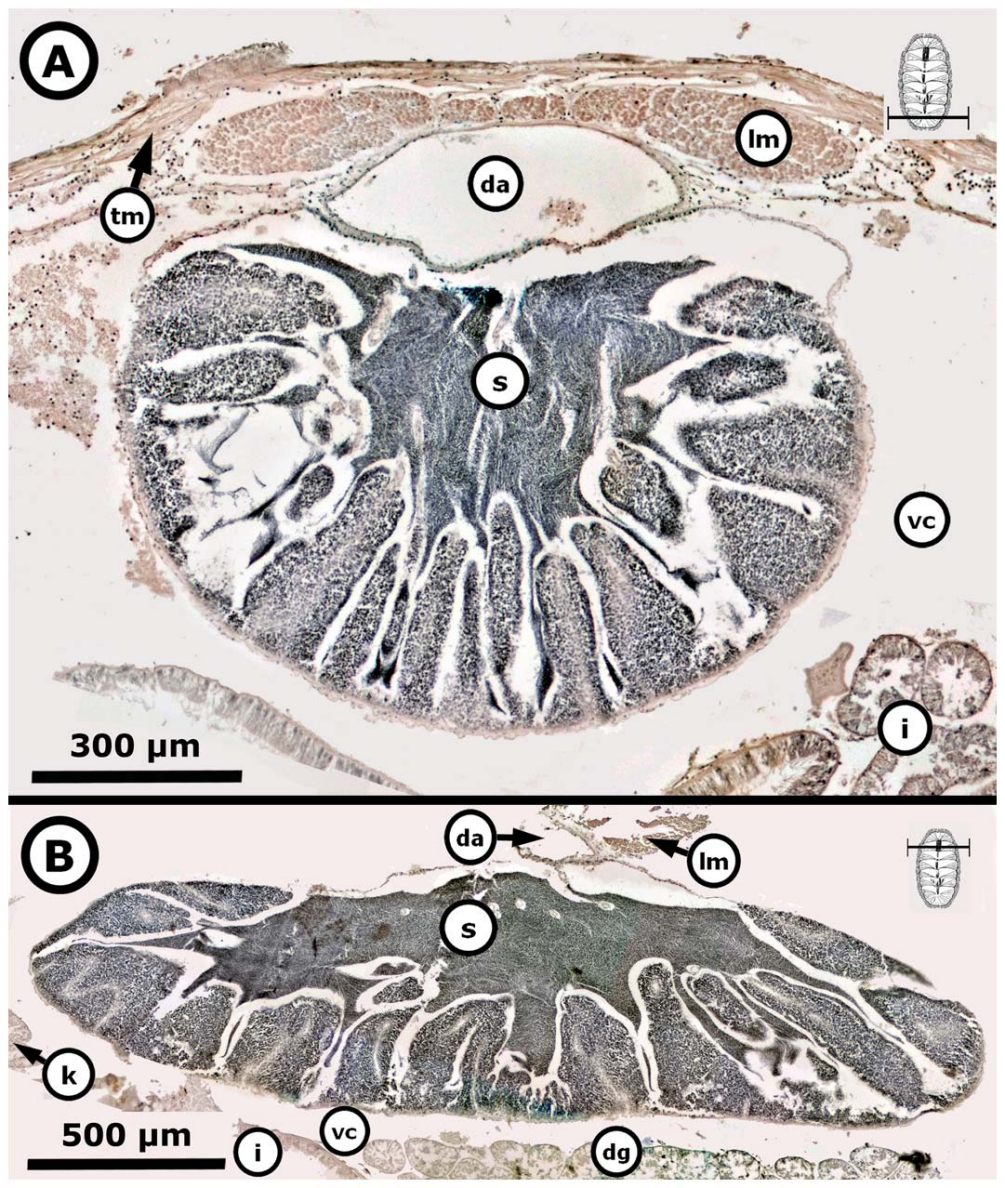
Mature *Chiton* males. Composite digital images in transverse section (**A**) of the posterior dorsal part and (**B**) of the anterior dorsal part from mature *Chiton albolineatus* males (A: 0.648 g BW, 25 mm TL, and 13 mm TW, and B: 1.365 g BW, 29.5 mm TL, and 16 mm TW) showing the gonadal lumen fully replenished with sperm. Abbreviations: da, dorsal aorta; dg, digestive gland; i, intestine; k, kidney; lm, longitudinal muscle; s, spermatozoa; tm, transversal muscle; vc, visceral cavity “hemocoel”.

Mature, i.e., period of formation of mature ova or sperm. Females of both *Chiton* spp. showed only a synchronous group of mature ova without replenishment by earlier oocytes (Fig. 15); with a confidence interval in TL of *P* (31.09 mm≤*μ*≤40.24 mm) for *C. articulatus* and of *P* (24.48 mm≤*μ*≤38.85 mm) for *C. albolineatus* (Fig. 4, S2-females). The mature ova, excluding the chorion, measured from 134 to 216 µm in diameter, and were characterised by an acidophilic cytoplasm filled with uniform protein yolk granules (vitellum) and a fully developed chorionic process with projections (i.e., chorion or hull) covering the mature ova and/or shed into the gonadal lumen (Fig. 15). Males reached the mature stage at a smaller size than females, with a confidence interval in TL of *P* (25.45 mm≤*μ*≤31.75 mm) for *C. articulatus* and of *P* (26.05 mm≤*μ*≤32.87 mm) for *C. albolineatus* (Fig. 4, S2-males), showing the gonadal lumen fully replenished with sperm (Fig. 16).

### Sexual differentiation (dimorphism) and onset of the first sexual maturity

The aforementioned histological examinations, as well as the ranges and confidence intervals among stages, indicated that the sexual differentiation (i.e., dimorphism, from the viewpoint of microscopic gonadal differentiation, since differentiable external traits are not present at this time, either in the gonad or in the anatomy of the organism) of *Chiton articulatus* and *Chiton albolineatus* occurs first as a female then as a male; although, males reach the mature stage earlier than females (Fig. 4). Thus, it would be possible to designate a sexual differentiation (i.e., gametogonia differentiation, stage 0: gametocytogenesis) at around 21.93 mm±1.16 mm (mean ± S.E.) in total length “TL” for *C. articulatus*, and at 21.79 mm±1.23 mm (mean ± S.E.) in TL for *C. albolineatus*.

The onset of the first sexual maturity (i.e., first time to a given size that each sex showed mature gametes (S2), such as ova filled with yolk granules and/or spermatozoa) for *C. articulatus* was around 32 mm in total length “TL” for females, and around 17 mm TL for males; while for *C. albolineatus* it was around 28 mm TL for females and 23.5 mm TL for males. Finally, males of *C. articulatus* mature before those of *C. albolineatus*; in contrast, females of *C. albolineatus* mature before those of *C. articulatus* (Fig. 4).

## Discussion

### Allometry

The b-values obtained here for *Chiton articulatus* and *Chiton albolineatus* were above three, demonstrating that these species have a standard isometric growth (i.e., growth that occurs at the same rate for all parts of an organism so that its shape is consistent throughout development) in total length “TL” and total width “TW” during their early life stages, i.e., from subjuvenile, through juvenile until subadult (4≤TL<40 mm). But just toward the adult stage (TL>40 mm), the b-values move downwards to between 2.9 and 2.2 for *C. articulatus* [43]–[45], and around 2.7 for *C. albolineatus* [47]. Therefore, this indicates that these species have first, during the early life stages, an isometric growth and subsequently an allometric growth (i.e., growth whereby parts of the same organism grow at different rates) as they develop into adulthood. Indeed, the allometric relationship with another chiton, *Lepidochitona cinerea*, has been variable through a full size range and has shown a change in the growth type, exhibiting b-values from 2.5 to 3.4 [61], [62].

Avila-Poveda [43] indicated that these changes in growth type occur because chitons subtly change their body shape, which is due to changes in the dorsal elevation of body height [63]. These changes in body shape are given by a direct relation with development and the physiological adjustment of the internal organs, but particularly evolve in response to gonad maturation. Therefore, gonads near maturity grow considerably, forcing the valves to bulge, making them more convex and leading to a more oval body shape. This also is supported by observations during dissections of mature gonads, since the gonad compresses the ventral organs (i.e., visceral mass) toward the foot to the point of almost merging the gonad with the visceral mass, making them difficult to separate [Abadia-Chanona and Avila-Poveda, pers. obs.].

Connors et al. [64] indicated that body shape is a biomechanical consequence in response to a passive and defensive conformation of the shell plate assembly, and a geometric enhancement of the structural assembly of the chitons to give mechanical robustness, which finally results in an approximately continuous curvature and a constant armor thickness that leads to a balance between mobility and protection. Therefore, the results of any allometric analyses in polyplacophorans are potentially subjected to increased variability due to differential growth rates of the different components during reproduction and to the flexibility of the mantle girdle [57], [58], so it would be essential in this type of analysis to perform them according to the early life stages (e.g., subjuvenile “post-metamorphosed”, juvenile, subadult, adult).

### Early life stages

Conspicuously, much of the literature on reproductive issues does not indicate a life stage for the organisms investigated, and instead they have been assumed to be adults and/or is limited to summarise that specimens were collected mainly during their reproductively active period, but the question is when do they start to be adults? This matter, in chitons, lacks consensus in the literature on the appropriate terminology and size to designate the “early life stages” for polyplacophorans. Hence, some size ranges are proposed to distinguish growth sequences or “early life stages” in these chitons, *Chiton articulatus* and *Chiton albolineatus*, according to gonadal ontogenesis, gonad development stages, sexual differentiation, and onset of the first sexual maturity here evaluated.

**Figure 17:**
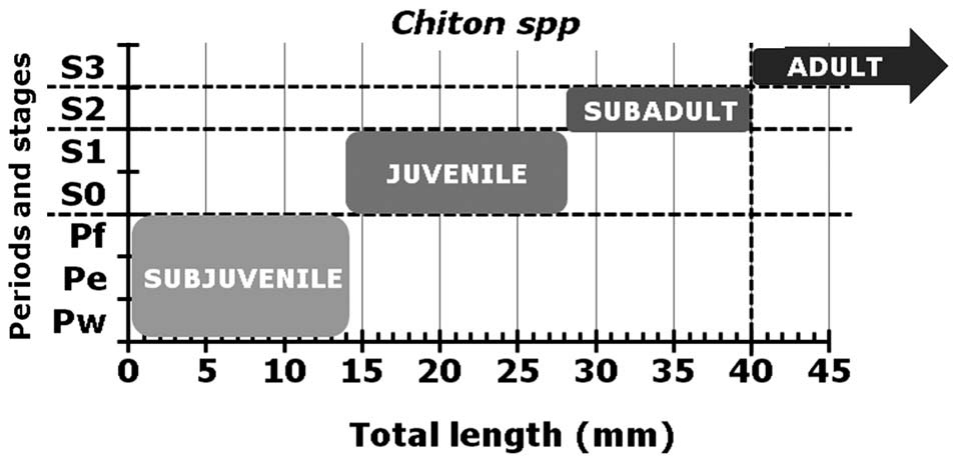
Early life stages for *Chiton spp*. Ranges in total length proposed to distinguish growth sequences or “early life stages” in the studied chitons, *Chiton articulatus* and *Chiton albolineatus*.

The term subjuvenile or post-metamorphosed defines the first growth stage that covers the criteria across all periods of gonad formation until a gonadal sac is built and expanded (i.e., Pw, Pe, Pf), without actually forming gametogonia or gametocytes. Particularly for *C. articulatus* and *C. albolineatus*, this stage was established at TL≤14 mm, i.e., until just before the lowest total length “TL” of the subsequent stage-0 female was observed (Fig. 17).

The term juvenile introduces young chitons that meet the criteria from Stage 0 (gametocytogenesis) and Stage 1 (gametogenesis) and is defined as the stage between the subjuvenile and subadult stages. Thus, it covers from the formation of oocytes or spermatocytes, both primary (i.e., diploid gametes) until the formation of oocytes or spermatocytes, both secondary, which include spermatids (i.e., first haploid gametes), but without actually forming mature gametes (i.e., ova and spermatozoa). For *C. articulatus* and *C. albolineatus*, this stage was established based on females between 14≤TL≤28 mm, i.e., between the lowest value of stage-0 and the lowest value of stage-2, in total length (Fig. 17).

The term subadult introduces chitons that, besides meeting criteria from Stage 0 and Stage 1 (particularly in males), also meet criteria from Stage 2 (mature), and is defined as the stage between the juvenile and the adult stages. The subadult stage commences with the full attainment of mature gametes (i.e., ova or spermatozoa), the so-called onset of first sexual maturity. For *C. articulatus* and *C. albolineatus*, this stage is very close and wholly superimposed (particularly in males) to the juvenile stage; thus, it is established based on females between 28≤TL≤40 mm, i.e., between the lowest value of stage-2 and the lowest value of stage-3, in total length (Fig. 17).

Finally, the adult stage commences after the subadult stage finalizes, then, for practical purposes, in *C. articulatus* and *C. albolineatus*, this stage was established at TL≥40 mm, and it included the future spawning stage-3 (Fig. 17).

Perhaps these stages are not adequate for all polyplacophorans, but they are at least for the family Chitonidae, which generally exhibits a common “adult”-size above 40 mm, including the chitons here studied [65], and also shows concordance with the modal size-class exhibited by other Mexican populations of both chitons [44]–[47].

### Sexual differentiation of the gonad

For molluscs [66], [67] and, particularly, from studies on Aplacophora [68], Polyplacophora [32], Bivalvia [69], Gastropoda [70], and Cephalopoda [49], [71]–[75], it has been indicated that the gonad remains undifferentiated throughout the larval life and their predetermined sex differentiation only appears during the post-metamorphic life. In effect, *Chiton articulatus* and *Chiton albolineatus* are invariably of separate sexes with a well-defined sexual differentiation that happens first as a female then as a male, during the beginning of the juvenile stage. Similar, to the polyplacophora [32]–[34], [37], as well as other mollusks, such as Aplacophora [68], Bivalvia [69], Gastropoda [70], and Cephalopoda [49], [71]–[75], it has been indicated that the sexual cells arise from specialised cells called protogonia, and emerge during the paired gonad primordium, but first as an ovary that can develop and suddenly as a testis. In fact, this is the timing within which the anatomical sexual phase is defined as ambisexual “hermaphroditism” or unisexual “gonochorism” [34], [66], [67].

Recently, it has been indicated that Chiton articulatus reveals an unusually high incidence of hermaphroditism, commonly being the female tissue which occupies the greatest part of the gonad sections respect to male tissue [76]. However, this study does not indicate the total length, “TL”, of the studied specimens. Therefore, it is difficult to integrate the information to recognize whether C. articulatus is: 1) a hermaphrodite species in the adult stage, and/or 2) whether these are just juvenile specimens who are in a process of sexual differentiation, which is more likely. Furthermore, a study of reproductive biology of C. articulatus from Oaxaca, Mexico is ongoing, and histological observations reveal not hermaphroditism for specimens of total length “TL” greater than 40 mm, i.e., adult stage [Abadia-Chanona, pers. obs.].

In this sense, results obtained in the present study indicate the practical possibility of recognising early, between 13≤TL≤20 mm, the sex of the chitons *Chiton articulatus* and *Chiton albolineatus*, i.e., between the lowest values of S0-both sexes, in total length, and during the start of the juvenile stage, which for practical purposes also corresponds to a medium size (i.e., 20 mm TL) in regard to the smallest adult size (i.e., 40 mm TL). Other chitons, holding essentially the same relations as those described here, reach sexual differentiation early, such as *Trachydermon raymondi* at around 5 mm TL [34]; *Lepidochitona cinerea* (as *L. cinereus*) females at 6 mm in total length “TL”, whereas males at 4 mm TL [36]; and *Stenoplax conspicua* females at 11.8 mm in total width “TW”, whereas males at 9.4 mm TW [37].

### Onset of the first sexual maturity

As a general rule for molluscs, the male sexual cells mature before the female sexual cells, whereby the initial phase of functional sexuality will be as a male [66], [67]. Here, for *Chiton articulatus* and *Chiton albolineatus*, this rule is followed, similar to that in the chitons *Lepidochitona cinerea* [36, as *L. cinereus*] and *Stenoplax conspicua* [37]. It is logical that males mature before females, as they do not need to accumulate reserve substances to feed the future embryo [25]–[27]. The female synthesises yolk “vitellogenesis” via nutrients deposited in the oocyte [2], [9]–[12], hence, the onset of the first sexual maturity becomes a critical event that triggers several physiological processes [77].

Based on our results, it is evident that chiton males went from gametocytogenesis (i.e., period of formation of early oocytes or early spermatocytes, i.e., immature gametes) toward a mature stage very quickly, suggesting that males from *Chiton articulatus* and *Chiton albolineatus* show a precocious beginning of the juvenile stage, which might suggest that they remain in the reproductive condition for a prolonged time and, consequently, they have the potential to expel their gametes in more than one occasion during their life cycle.

## Summary/Conclusion

Overall, we conclude that *Chiton articulatus* and *Chiton albolineatus* have during the early life stages (i.e., from subjuvenile, through juvenile until subadult: TL<40 mm), an isometric growth and, subsequently, an allometric growth as they develop into adulthood (TL>40 mm). So it would be essential to conduct this type of analysis considering the early life stages (i.e., subjuvenile “post-metamorphosed”, juvenile, subadult, adult). The end of the subjuvenile stage (post-metamorphic) is characterised by the beginning of gonadal ontogenesis (i.e., gonad emergence, paired formation of the gonadal sac, the merge, development, and finally expanding of the gonad sac), although gonadal morphogenesis continues to happen during the juvenile stage but this time with the sexual differentiation and onset of the first sexual maturity. The size range designed to each “early life stage” is a good indicator of growth sequences of these two chitons since they express stages according to gonadal ontogenesis, gonad development stages, sexual differentiation, and onset of the first sexual maturity here evaluated. *Chiton articulatus* and *Chiton albolineatus* are invariably of separate sexes with a well-defined sexual differentiation that happens first as a female then as a male; although, males reach the mature stage earlier than females, thus, revealing the practical possibility of recognising the sex and the onset of the first sexual maturity during the juvenile stage of the chitons, thus, for practical purposes also corresponds to a medium size (i.e., 20 mm TL) in regard to the smallest adult size (i.e., 40 mm TL).
